# Trabecular bone anisotropy imaging with a compact laser-undulator synchrotron x-ray source

**DOI:** 10.1038/s41598-017-14830-x

**Published:** 2017-11-03

**Authors:** Christoph Jud, Eva Braig, Martin Dierolf, Elena Eggl, Benedikt Günther, Klaus Achterhold, Bernhard Gleich, Ernst Rummeny, Peter Noël, Franz Pfeiffer, Daniela Muenzel

**Affiliations:** 1 Technical University of Munich, Chair of Biomedical Physics, Department of Physics and Munich School of BioEngineering, Garching, 85748 Germany; 2 Technical University of Munich, Department of Diagnostic and Interventional Radiology, Klinikum rechts der Isar, Munich, 81675 Germany; 3 Max Planck Institute of Quantum Optics, Garching, 85748 Germany; 4 Technical University of Munich, Institute for Advanced Study, Garching, 85748 Germany

## Abstract

Conventional x-ray radiography is a well-established standard in diagnostic imaging of human bones. It reveals typical bony anatomy with a strong surrounding cortical bone and trabecular structure of the inner part. However, due to limited spatial resolution, x-ray radiography cannot provide information on the microstructure of the trabecular bone. Thus, microfractures without dislocation are often missed in initial radiographs, resulting in a lack or delay of adequate therapy. Here we show that x-ray vector radiography (XVR) can overcome this limitation and allows for a deeper insight into the microstructure with a radiation exposure comparable to standard radiography. XVR senses x-ray ultrasmall-angle scattering in addition to the attenuation contrast and thereby reveals the mean scattering strength, its degree of anisotropy and the orientation of scattering structures. Corresponding to the structural characteristics of bones, there is a homogenous mean scattering signal of the trabecular bone but the degree of anisotropy is strongly affected by variations in the trabecular structure providing more detailed information on the bone microstructure. The measurements were performed at the Munich Compact Light Source, a novel type of x-ray source based on inverse Compton scattering. This laboratory-sized source produces highly brilliant quasi-monochromatic x-rays with a tunable energy.

## Introduction

Conventional x-ray radiography is a standard diagnostic technique in clinical trauma imaging, providing a high diagnostic accuracy for fractures of the cancellous bones of the extremities. In Germany, 1.7 x-ray investigations per person and year are made^1^, but it is estimated that in such radiographs about 3.7% of all fractures in the extremities might be missed. Such radiographically occult or subtle fractures may delay the healing process or cause further complications^2–5^. If radiographs are indecisive, Magnetic Resonance Imaging can potentially lead to additional diagnostic information but x-ray based imaging such as CT can provide similar information and is generally more available^6^. One recently developed method that could improve radiography in the first place is grating-based x-ray darkfield-contrast (DFC) imaging, which is related to the ultrasmall-angle scattering properties of the specimen^7–10^. Using DFC imaging, Tanaka *et al*. investigated metacarpophalangeal joints of an *in vivo* human finger and detected cartilage in joints^11^, and Thuering *et al*. demonstrated improved diagnostic value of radiographs of human cadaver hands^12^.

Single DFC images, however, are only sensitive to one specific scattering direction and therefore could potentially miss information. Exploiting this directional dependence, Jensen *et al*. proposed the method of x-ray vector radiography (XVR)^13,14^. It combines several DFC-images with different sample orientations around the optical axis to a multi-contrast image. Since the optical gratings are symmetric, the signal dependence has a periodicity of 180°. Malecki *et al*. proposed a Lambert-Beer-like relation between the DFC-images and the scattering strength, which they found to have a sinusoidal dependence with respect to the rotation angle. From a fit curve, the mean scattering strength, anisotropy and the orientation of scattering structures can be extracted. For trabecular bone tissue, a correlation between XVR and the trabecular microstructure has been shown already^15–17^. A connection between the degree of anisotropy and femoral bone strength as well as an improved prediction of vertebral failure load indicate the potential of XVR for osteoporosis imaging^18,19^. However, all investigations so far have been performed with isolated samples of limited size. Here, we present an XVR of an *ex-vivo* human hand and thereby show the potential of this measurement technique in detecting clinically occult fractures, in particular when the anisotropy image is assessed for the diagnosis.

## Results

**Figure 1:**
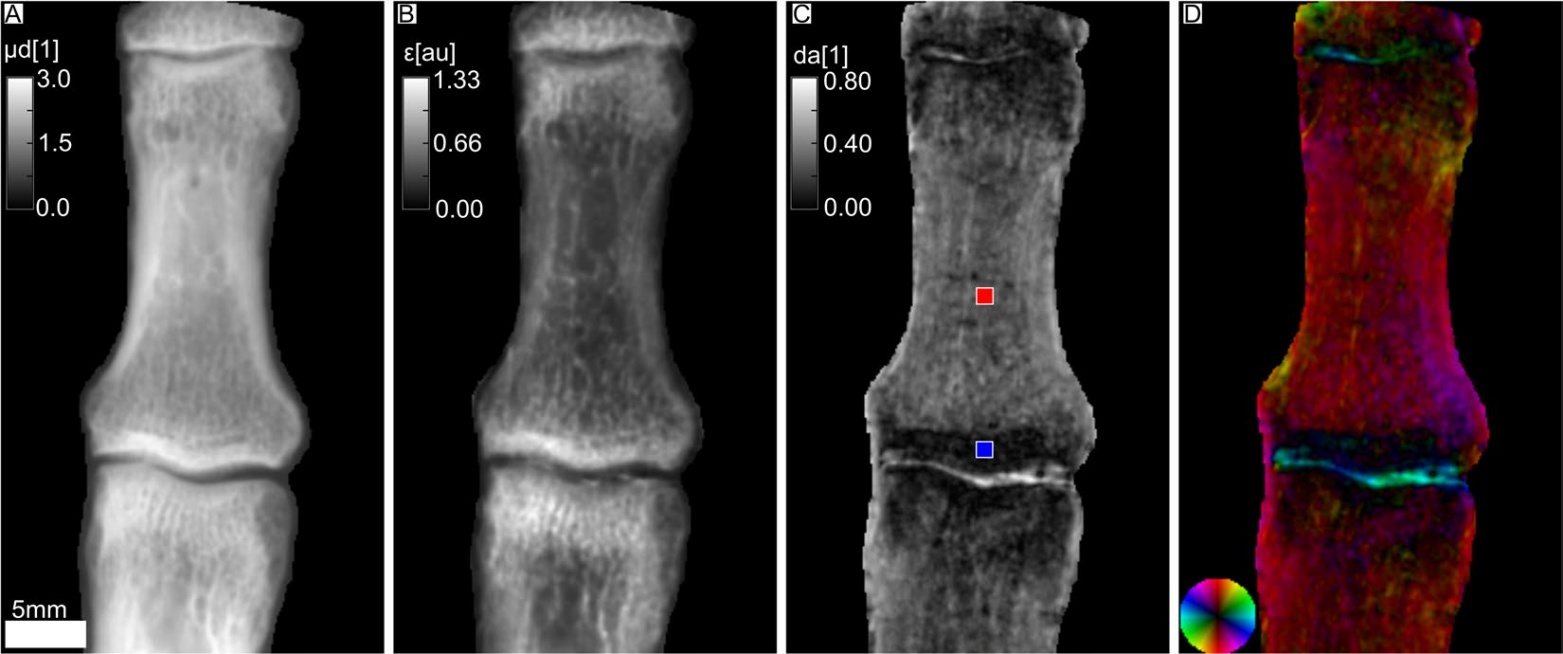
Attenuation and x-ray vector radiography (XVR) images of a human index finger. The head of the proximal phalanges, the intermediate phalanges and the base of the distal phalanges are visible. In (**A**) the integrated attenuation coefficient is shown and (**B**) depicts the mean scattering strength. (**C**) Shows the degree of anisotropy as defined in Eq. 1, i.e. the difference of maximum and minimum scattering divided by its sum. The mean values in the colored ROI’s are da_blue_ = 0.07 and da_red_ = 0.25 in the blue ROI and red ROI, respectively. (**D**) Presents the orientation of scattering structures color-coded according to the color wheel shown in the bottom left. The brightness corresponds to the degree of anisotropy.

In Fig. 1, the index finger of an *ex-vivo* human hand is depicted. The intermediate phalanx is visible as well as parts of the distal phalanx and the proximal phalanx. The integrated attenuation coefficient illustrates the typical anatomy of the long bones with outlining cortical bone and internal trabecular structure (see Fig. 1A). Parts B, C and D illustrate the XVR. In B, the mean scattering signal averaged over different sample orientations is shown. It mainly corresponds to ultra-small-angle scattering at interfaces and sub-micron structures in the bone^20,21^. The mean scattering strength is lowest in the central part of the diaphysis, slightly increasing towards the outside. It has its highest value in the epiphysis, close to the joint. The degree of anisotropy, defined as

1 $${\rm{da}}=\frac{{\rm{\max }}.{\rm{scattering}}-\,{\rm{\min }}.{\rm{scattering}}}{{\rm{\max }}.{\rm{scattering}}+\,{\rm{\min }}.{\rm{scattering}}},$$

is lowest in the epiphysis with a mean value of da_blue_ = 0.07 (blue ROI) and higher in the diaphysis with a mean value of da_red_ = 0.25 (red ROI). Its highest value is found at the transitions from the bone to the liquid-filled joint. A value of zero thereby indicates completely isotropic scattering (same scattering in all directions) and a value of one indicates perfectly anisotropic scattering (no scattering in one direction and maximal scattering in the orthogonal direction). The orientation of scattering structures is illustrated in D, it is orthogonal to the direction of maximal scattering. A distinct color corresponds to an orientation in between [0°, 180°] represented by the color wheel in the bottom left, the brightness encodes the degree of anisotropy already shown in C. It illustrates that the scattering structures are predominantly oriented in the longitudinal direction. The highly anisotropic signal at the transition between bone and joint is oriented parallel to the bone surface.

**Figure 2:**
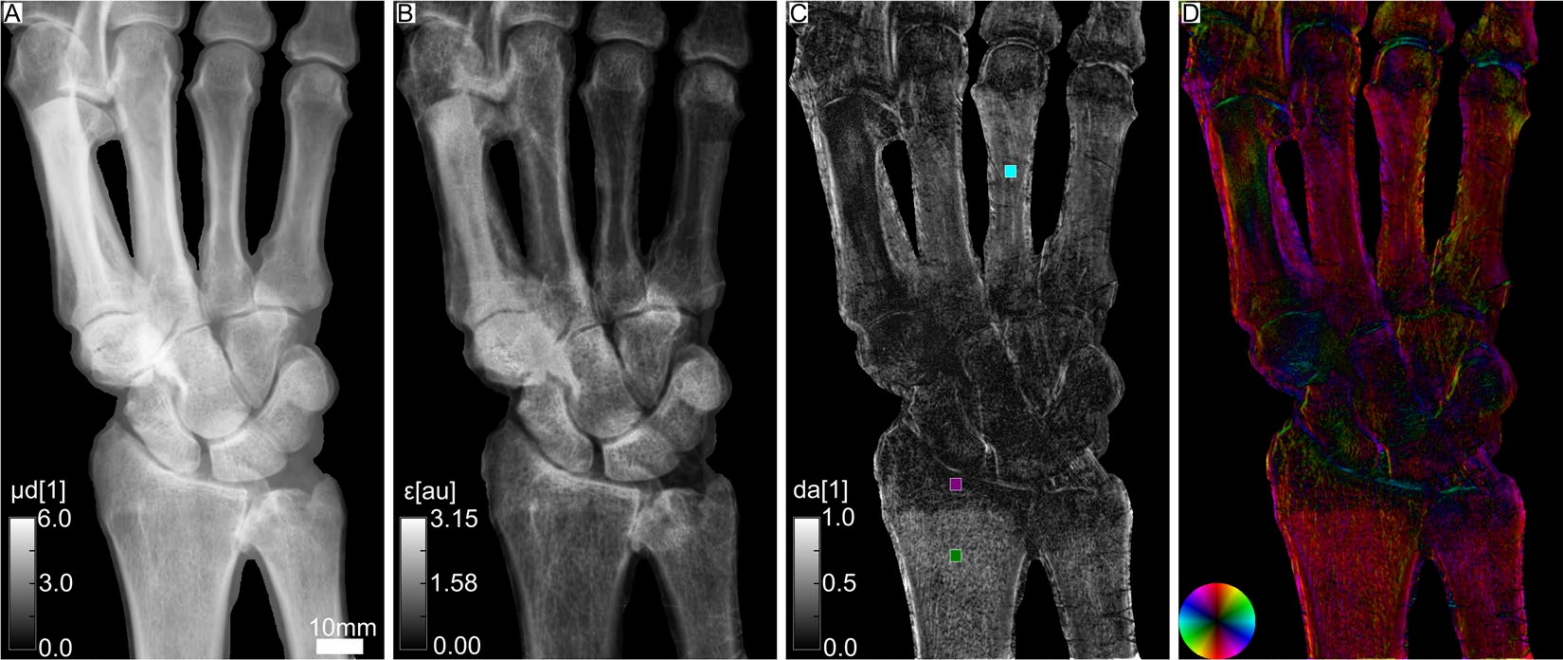
Attenuation and XVR images of a human hand, showing the radius, ulna, carpals and metacarpals. In (**A**) the integrated attenuation coefficient is depicted. (**B**) Depicts the mean scattering strength. (**C**) Illustrates the degree of anisotropy, i.e. the difference of maximum and minimum scattering divided by its sum. The mean values in the colored ROI’s are da_cyan_ = 0.27, da_purple_ = 0.08 and da_green_ = 0.41. In (**D**) the orientation of scattering structures is color-coded according to the color wheel shown in the bottom left. Brightness once again corresponds to the degree of anisotropy.

**Figure 3:**
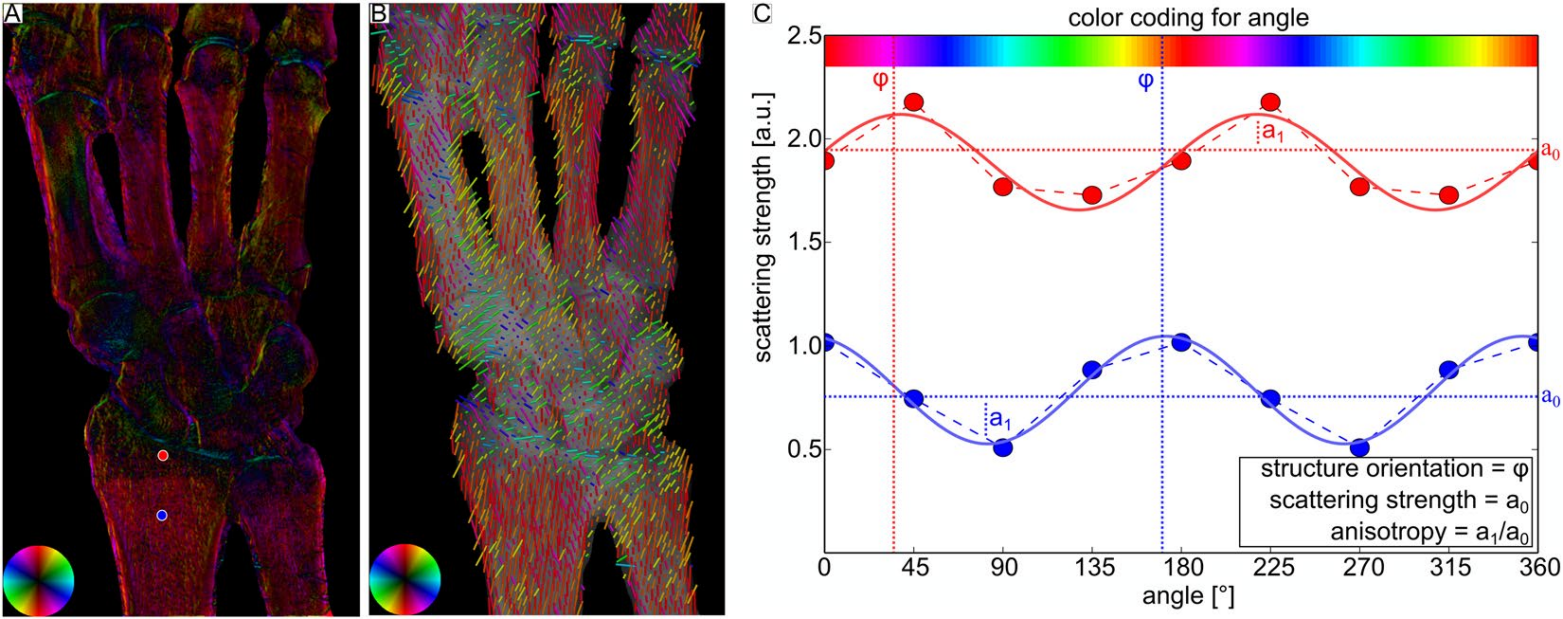
Illustration of how the XVR-data is extracted from different dark-field contrast (DFC) images. In (**A**) the orientation of scattering structures is shown color-coded, the brightness corresponds to the degree of anisotropy. (**B**) Illustrates an alternative representation using a vector field. Their color emphasizes the direction, the degree of anisotropy is encoded by the length. (**C**) Depicts the scattering strength versus the sample orientation relative to the grating interferometer for two pixels marked by a red dot and a blue dot in A. From the sinusoidal fit, a_0_ corresponds to the mean scattering, the phase *ϕ* corresponds to the angle of maximal scattering and the ratio a_1_/a_0_ to the degree of anisotropy.

Figure 2 shows results for an *ex-vivo* human hand. Anatomically, its bones can be categorized as long bones as the metacarpals, radius, ulna, or short bones, including all bones of the carpus. Analogue to Fig. 1, four different contrast modalities are illustrated. In the attenuation image A, one can see that the index finger covers the thumb. Therefore, the x-ray beam did not fully penetrate the sample in this region and hence it contains less information. The mean scattering signal is depicted in B. There is no obvious difference of scattering signal between the different parts of the bones. However, the radius and ulna show a very pronounced change in the degree of anisotropy, as can be seen in C. The epiphysis has a low degree of anisotropy of da_purple_ = 0.08. In the diaphysis, it is considerably higher with a mean value of da_green_ = 0.41 in the green ROI. The metacarpal bones have a degree of anisotropy of da_cyan_ = 0.27, which is comparable to the intermediate phalanges shown in Fig. 1C. All carpals have a homogeneous and low degree of anisotropy. As already observed in Fig. 1C, a high degree of anisotropy is found right at the transition from bone to the joint. In D, the orientation of scattering structures is depicted. The carpals do not show any preferred orientation of scattering structures in contrast to radius, ulna and the metacarpal bones which have longitudinally oriented structures. However, the orientational preference becomes less pronounced in regions with low degree of anisotropy. In all joints, there are scattering structures oriented along the bone edge similar to Fig. 1D. Figure 3 shows an alternative visualization of the XVR-data. For comparison, part A again depicts Fig. 2D. In B, the same information is depicted as a vector field. The direction indicates the orientation of scattering structures, additionally color coded corresponding to the color wheel. The degree of anisotropy is given by the vector length. Note that in contrast to A, only a limited number of data points can be shown. XVR exploits the scattering dependence on the sample orientation relative to the grating interferometer. In C, the dependence of the scattering strangth on the rotation around the optical axis is given for two pixels marked by a red dot and a blue dot in A. The measured data is shown twice to emphasize the periodicity of 180°. From the fit, the mean scattering signal can be deduced as the offset value a_0_, the phase *ϕ* corresponds to the angle of maximal scattering. For instance, a phase of zero indicates maximal scattering in the horizontal plane and hence an orientation of scattering structures in the vertical plane, corresponding to a red color on the color wheel. According to Eq. 1, the degree of anisotropy is given by the ratio *a*_1_/*a*_0_.

## Discussion

Conventional x-ray radiography is an established tool in clinical imaging and is widely used to image fractures of the trabecular bones of the extremities. However, non-displaced fractures and microfractures of the trabecular bone are often not visible in conventional radiography, resulting in a lack or delay of adequate therapy.

In contrast to attenuation, the mean scattering provided by XVR is sensitive to ultrasmall-angle scattering and hence to sample morphology. The bony structure mainly consists of trabecular spongy matrix with multiple interfaces, which are responsible for a high scattering effect on the x-ray beam. Cortical bone consists of a dense arrangement of multiple cylindrical units, so-called osteons. Each osteon has a central canal which represents a vascular channel for bone nutrition. Thus, multiple interfaces between single osteons with central canals leads to a high scattering signal of cortical bone. All of this results in a homogeneous mean scattering signal of the epiphysis and diaphysis in the long bones and within the carpals. Variations in the mean scattering signal correlate to the variations in the attenuation contrast and are therefore most likely due to density and thickness variations.

The XVR signal, on the other hand, shows a clear difference of the degree of anisotropy within the different parts of the long bones, corresponding to the microstructure of the bony anatomy. The osteons within the cortical bone are arranged in a linear order, lying parallel to each other and parallel to the long axis of the long bones^22^. In the diaphysis, the orientation of the trabecular bone is mainly lamellar as well, with a reduction of the total bone mass due to the spongious structure. Linear orientation of the osseous mineralized microstructure of cortical bone and trabecular bone of the diaphysis of long bones result in high anisotropy with a longitudinal internal orientation. In contrast, the trabecular bone within the epiphysis shows no linear but radial orientation of the bony microstructure. The growth process of the epiphysis follows a radial orientation of the cell columns, resulting in a radial orientation of the trabecular structure of the mature bone^22^. As a result, epiphyseal areas show a low degree of anisotropy with divergent orientation of bony microstructures, explaining the different quantitative values observed in Figs 1 and 2. The sharp transition between anisotropic scattering and the isotropic one consequently corresponds to the epiphyseal line which divides the diaphysis from the epiphysis. Short bones as the carpalia do not have longitudinal orientation but consist of trabecular structure without any orientational preference. This results in a low degree of anisotropy, as can be seen in Fig. 2. The anisotropic signal oriented along the joint surface is caused by the transition between the bone tissue and the liquid filling the joints. Such interfaces on a scale below the spatial resolution lead to a scattering signal in orthogonal direction to the surface and hence the detected orientation follows the joint surface.

Microfractures with a resulting discontinuity of the trabecula and included hematoma will result in a loss of the anisotropic scattering signal in XVR. Therefore, in acute trauma diagnostics, XVR may help to increase sensitivity in so-called radiographically occult fractures of the trabecular bones.

However, several limitations have to be addressed in order to clinically apply this technique. The energy of 25 keV should be increased in order to reduce beam starvation and dose. In addition, the number of phase steps required for one DFC-image should be reduced as well as the number of different sample orientations to a minimum of three points necessary to fit a sinusoidal curve of known period. Another approach could be a so-called single shot technique, requiring that the fringes are directly resolved by the detector. It has been shown that this technique can reveal DFC-images for *in-vivo* objects with a dose comparable to conventional chest radiographs^23–25^. In contrast to conventional radiography, XVR detects subpixel-sized information. This could allow a reduction of spatial resolution and lead to a decreased dose level as well. Recently developed phase grating far-field interferometers could avoid absorption gratings and thus further reduce the dose^26–28^. For mammography applications, it has already been shown that a Talbot interferometer can get equal contrast in attenuation as a conventional setup at a comparable dose level^29^. In order to reduce the measurement time, the field of view should be increased in order to fully cover a single hand within one acquisition. The MuCLS is a well-suited source for XVR since the high flux reduces the acquisition time as well. The quasi-monochromatic radiation mostly avoids beam-hardening artifacts and could allow to further reduce the dose with respect to a polychromatic setup^30^. However, XVR has been successfully applied in laboratory setups as well, showing the feasibility of clinical application^18,19^.

In conclusion, XVR provides a suitable tool for directly detecting the anisotropy and orientation of scattering structures. It is sensitive to the bone microstructure and thereby has the potential to improve the diagnostics of so-called radiographically occult fractures.

## Methods

**Figure 4:**
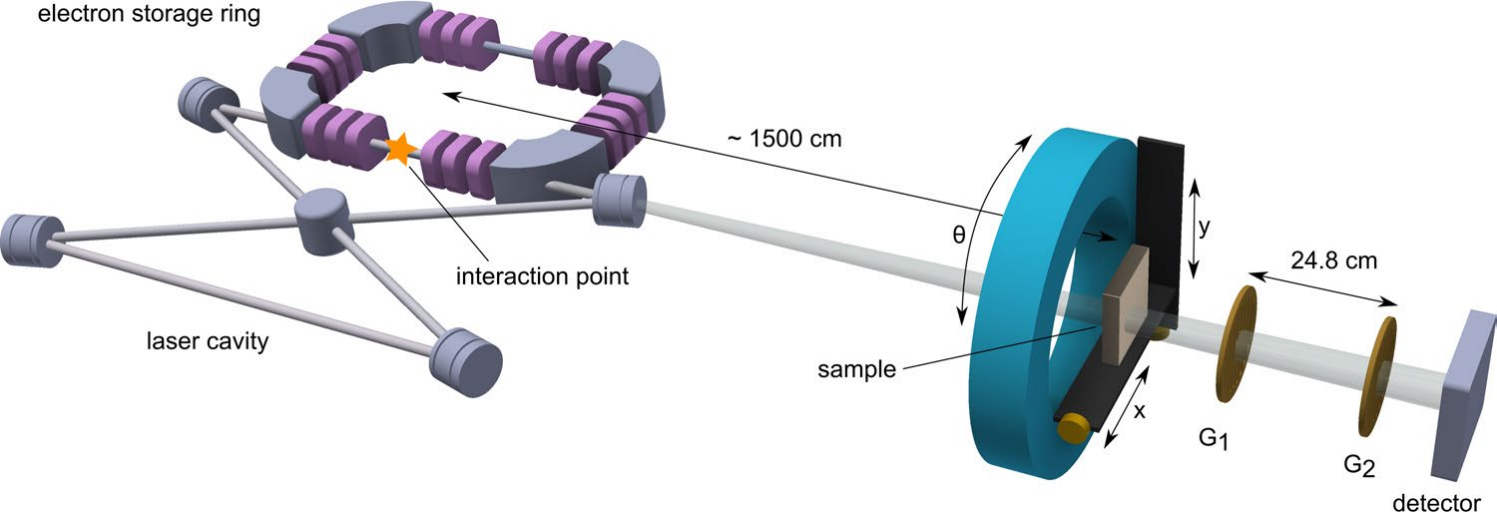
Scheme of the experimental setup. To the left, the Munich Compact Light Source (MuCLS) with electron storage ring and laser cavity is visible. X-rays are generated in the interaction point. The sample is placed approximately 15 m downstream from the source, a Talbot grating interferometer allows for XVR imaging. Note that the proportions are strongly exaggerated.

The sample was obtained from a human body donor dedicated to the medical school dissection courses at the Institute of Anatomy (Ludwig-Maximilian-University of Munich). The human body donor had given written consent to provide the body after deceasing for medical education and research according to international ethical guidelines and according to German law. The *ex-vivo* human hand specimen was fixed in formalin. During the measurement, the sample was embedded into a plastic bag and fixed within a dedicated sample holder. The measurement was performed at the Munich Compact Light Source (MuCLS) in Garching, Germany, a novel type of x-ray source which uses the effect of inverse Compton-scattering to produce highly brilliant quasi-monochromatic x-rays^31–38^. It mainly consists of a small electron storage ring with tunable energies between 25 and 45 MeV and a laser cavity, as illustrated in Fig. 4. The high-finesse laser cavity resonantly stores an infrared laser (*λ* = 1064 nm), the stored power is about 70 kW. In the interaction point, focused laser bunches collide with electron bunches in an area of 42 × 42 *μ*m^2^. The repetition rate of about 65 MHz yields a flux of about 0.97 · 10^10^ photons/s and a brilliance of 4.8 · 10^9^ photons/s mm^2^ mrad^2^ 0.1%BW^37^. The spectrum is similar to an undulator spectrum with fundamental wavelength *λ*/(4*γ* ^2^), where *λ* is the laser wavelength and *γ* the electron energy over its rest mass. By tuning the electron energy, one can therefore select x-ray energies within 15–35 keV. For this experiment, an x-ray energy of 25 keV was used. The experimental hutch was situated about 15 m downstream from the interaction point. With a divergence angle of 4 mrad, the beam had a diameter of about 60 mm. The Talbot interferometer consisted of a phase grating (G_1_) with a period of 4.92 *μ*m and a duty cycle of 0.5. Nickel with a height of 4.39 *μ*m provided a phase shift of *π*/2 at the design energy of 25 keV. The absorption grating G_2_ was placed 248 mm downstream G_1_ in the first fractional Talbot distance. It had a period of 5 *μ*m with a duty cycle of 0.5 and consisted of Au of more than 70 *μ*m height. The detector was a Varian PaxScan 2520DX with a GadOx scintillator and a pixel size of 127 × 127 *μ*m^2^. For the XVR measurement, a stitched image consisting of 5 × 5 single phase-contrast projections was generated for four different sample rotations around the optical axis with angles *θ* = {0°, 45°, 90°, 135°}. For a single phase-contrast projection, 7 phase steps were acquired with an exposure time from 0.5 s to 10 s, depending on sample thickness. In the next step, all stitched images are registered such that the scattering strength depending on *θ* can be analyzed. A sinusoidal fit with a given period of 180° yields the offset value *a*_0_, amplitude *a*_1_ and phase *ϕ*, which corresponds to the direction of maximal scattering (cf. Fig. 3).
